# The Effect of Feed Frequency on Growth, Survival and Behaviour of Juvenile Spiny Lobster (*Panulirus ornatus*)

**DOI:** 10.3390/ani12172241

**Published:** 2022-08-30

**Authors:** Katarzyna Kropielnicka-Kruk, Quinn P. Fitzgibbon, Basseer M. Codabaccus, Andrew J. Trotter, Dean R. Giosio, Chris G. Carter, Gregory G. Smith

**Affiliations:** Institute for Marine & Antarctic Studies (IMAS), University of Tasmania, Private Bag 49, Hobart, TAS 7001, Australia

**Keywords:** aquaculture, crustacean, behaviour, cannibalism, nutrition, tropical rock lobster

## Abstract

**Simple Summary:**

Sensory stimuli including olfactory, visual, acoustic and tactile input, are important aspects of feed exposure and optimisation of feeding schedules. These aspects were investigated in our study, with a focus on the effect of feed frequency (one, two, four, eight, sixteen and thirty-two feed supplies per day) on survival, growth and behaviour of juvenile P. ornatus. Increasing daily feed frequency from one to sixteen improved feed intake and growth.

**Abstract:**

Spiny lobsters have a range of complex chemical communication pathways that contribute to feeding behaviour. Feed intake is modulated by feed availability and feed characteristics, such as attractiveness and palatability, with behavioural factors, such as social competition and circadian rhythm, providing an extra layer of complexity. In this study, we investigated the effect of feed frequency on survival and growth of early-stage (instar 2–6) juvenile *Palunirus ornatus*. In addition, we investigated the interactive effect of feed frequency and circadian rhythm on lobster feed response. Lobsters were fed a set ration at a frequency of either one, two, four, eight, sixteen or thirty-two times per day over 49 days. The effect of feed frequency on growth and survival was determined. Circadian feeding activity under these feeding treatments was assessed by time-lapse photography. Increased feed frequency from one to sixteen feeds daily improved growth by increasing apparent feed intake (AFI) and feed attraction, as confirmed by the increased presence of lobsters in the feeding area. The rapid leaching of feed attractant, particularly free amino acid, suggests a beneficial effect of multiple feeding frequencies on feed intake and growth. However, more than sixteen feeds per day resulted in decreased feed intake and a subsequent reduction in growth. The decrease in feed intake is thought to be associated with saturation of the culture environment with attractants, resulting in a reduced behavioural response to feed supplies. This may indicate the need for depletion of attractants to retrigger a feeding response. As lobsters were grown communally, faster growth at sixteen rations per day was also coupled with increased cannibalism, likely driven by increased vulnerability with the occurrence of more frequent ecdysis events. Whereas circadian rhythm indicated more activity at night, an interaction between daytime activity and feed frequency was not observed.

## 1. Introduction

Effective nutrient intake is key to the nutrition of any organism and is critical to achieving optimal fitness and growth in the wild and under aquaculture conditions. Several factors are involved in ensuring that lobster feed intake occurs, including location of chemical feed signals, feed perception, identification, attractiveness, motivation, feed capture and ingestion [1,2]. These steps are initiated through the stimulation of the olfactory system (located on antennules) and distributed (located on all body areas) chemoreception [2,3]. The antennular chemoreceptors are used to search for and locate feed, whereas chemoreceptors located on the legs and mouthparts provide information that allows the lobster to catch and consume or reject feed [1,2]. Chemoreceptors in crustaceans are often specialised to detect specific substances and allow for detection of key components among complex mixtures [4]. The behavioural response to the presence of attractants is usually stronger for complex mixtures, indicating a synergistic effect; however, single chemicals, such as glycine, have been shown to provide a strong attraction for the spiny lobster (*Panulirus interruptus*) [4,5]. Feeding can be accompanied by learned behaviours, which can influence the decision as to whether a feed search should be initiated [6]. In the wild, spiny lobsters exhibit omnivorous and detritivore feeding, consuming a range of foods, including crustaceans, gastropods and marine plants [7,8,9]. The presence of feed also triggers agonistic behaviours, including competition and dominance, and may, as a consequence, lead to variable growth rates within an aquaculture cohort [10,11]. 

At the Institute for Marine and Antarctic Studies (IMAS), hatchery technologies have been developed for the tropical rock lobster, *Panulirus ornatus* [12,13,14]. The development of a formulated feed is key to the development of a commercial lobster grow-out industry. Feed attractiveness is a major component of feed intake the subsequent development of a formulated feed for spiny lobsters. Low-molecular-weight compounds, such as free amino acids (e.g., glycine), betaine and organic acids, are known to provide attraction to feeds [15,16]. However, these same compounds are water soluble and leach rapidly from the feed when submerged, thus providing a short window during which feeds are most attractive. Marchese et al. [17] demonstrated that formulated feeds were attractive to lobsters for 2-3 hours after supply, whereas mussels remained attractive throughout the daily feeding cycle. Increasing the feed frequency of set rations to extend the time that feeds remain attractive is one strategy that has been investigated for several crustacean species. The exposure of juvenile prawns *Penaeus merguiensis*, *Penaeus vannamei* and *Penaeus monodon* to different feeding frequencies showed the highest growth in groups fed most frequently, which was four (for *P. merguiensis* and *P. vannamei*) or six (for *P. monodon*) times per day [18,19,20]. However, a number of studies involving spiny lobsters have demonstrated that increased feed frequency did not improve growth [11,21,22,23]. Syafrizal et al. [22], found inconclusive results with increased feeding frequency on the growth in juvenile *Panulirus versicolor*, while Thomas et al. [11] noted decreased competitive and agonistic behaviours in *Jasus edwardsii* with increased feeding frequency. Cox and Davis [23] found that feeding *Panulirus argus* juveniles once a day to excess at the start of the dark phase was beneficial. Thus, in the present study, our primary aim was to investigate an optimal feed frequency for maximising growth and survival. In addition, we verified whether response to feed is an interactive effect between feed frequency and circadian rhythm.

## 2. Materials and Methods

### 2.1. Experimental Animals, Design and Culture System

Juvenile lobsters were hatchery-reared from eggs at IMAS Taroona, University of Tasmania, based on previously described methods [12,13,24]. Before experimental allocation, juvenile lobsters were co-fed with shucked blue mussel flesh, moist feed and a commercial-in-confidence dry pellet (0.8 mm diameter). Juvenile lobsters (instars 2 to 6) of average initial carapace length (CL_0_, 9.8 mm ± 0.18) and initial wet weight (WW_0_, 0.93 g ± 0.05) were stocked randomly into the experiment vessels. Eleven lobsters were allocated to each replicate; the average initial biomass (B_0_) per replicate was 10.6 g ± 0.01. Six feeding frequencies were tested: one (F1), two (F2), four (F4), eight (F8), sixteen (F16) and thirty-two (F32) feed portions per day. The daily feeding regime extended over twenty-two hours per day, with the remaining two hours per day reserved for waste removal (siphoning) and system maintenance. The daily feeding time interval within each frequency was the same for all replicates. Treatments comprised six replicates (n = 6); however, one replicate from the F8 was lost due to system failure (n = 5). The replicate culture vessels were 18-L blue polypropylene tanks (38 cm long, 24 cm wide and 24.8 cm high) equipped with sixteen small hides per vessel (PVC tubes; 2.5 cm diameter × 5 cm length) with eight shelters located along one short wall of the vessel and eight along the long wall of the vessel plus two 10 cm mini-Mills mesh collectors [25] hanging off the adjacent long wall. After nine days, four medium hides (PVC tubes; 3 cm diameter × 6 cm length) were added to accommodate larger animals as they moulted. For the last nineteen days, hides consisted of three small, four medium and eight large (PVC tubes; 4 cm diameter × 8 cm length) hides and two mini-Mills mesh collectors. All tube hides used in the experiment were lined with 2 mm fibreglass fly mesh. The water supplied to the vessels was filtered to 10 µm and sterilized by ozonation to 725 mV for 10 min. Water quality physical parameters were maintained at 27.7 °C ± 0.03, pH 8.10 ± 0.01 salinity 33.7 ppt ± 0.03 and dissolved oxygen 117.4% sat. ± 0.58. Water exchange in the culture vessels was maintained at six turnovers by volume h^−1^. The photoperiod was 12:12 L:D, and light was supplied with Fluval Aquasky 2.0 LED lights. The red light was on for 24 h day^−1^, and during the light-phase, blue light was supplied for 12 h day^−1^. The red light that was used for improved visibility during time-lapse recordings, is not visible to lobsters and does not disturb their night activity [12,26,27]. The photoperiod, at 12:12 L:D, was in the range occurring in the natural environment and has previously been shown not to negatively affect feed intake, growth or survival of lobsters [28,29,30]. Moulting events and mortality were recorded every morning and prior to siphoning before the start of the next feeding cycle, with the work order randomised daily.

### 2.2. Experimental Feed and Feeding

**Figure 1:**
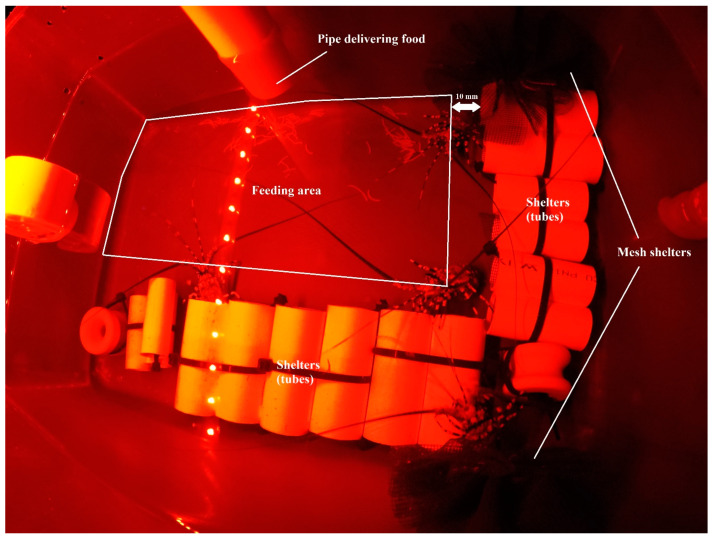
Plan of experimental vessel floor, showing distribution of shelters, location of pipe delivering food and feeding area.

Throughout the duration of the experiment, lobsters were fed solely on commercial-in-confidence dry pellet feed. The daily feed ration was set at 25% of lobster body weight. On day 23, lobsters from each replicate were bulk-weighed for group WW to adjust the feed ration. Daily rations were divided into equal portions among the tested feed frequencies. Feed portions for each frequency were supplied by FIAP belt feeders, which were calibrated to supply feed portions in set time intervals corresponding to the assigned frequency. Belt feeders were equipped with funnels and transfer PVC pipes to deliver the feed below the water surface and 10 cm above the vessel base (Figure 1). The feeding cycle began after commencement of the dark phase. Feed was delivered to a feeding area on the vessel floor. The feeding area was defined by one long and one short wall of the vessel and 10 mm distance from rows of shelters located alongside the opposite walls (Figure 1).

### 2.3. Apparent Feed Intake

The apparent feed intake (AFI) was recorded during the sixth week of the experiment [14]. For 7 days, feed waste was collected from all replicates after the twenty-two hours of the feeding cycle by siphoning into separate 124 µm sieves, rinsed with deionised water and frozen for later analysis. After completion of the experiment, feed rations were supplied to vessels with no lobsters present to account for the percentage dry matter loss in water. Samples of feed before and after submersion were dried at 105 °C for 24 h to determine dry weight (DW). The AFI was obtained by calculating the difference between the DW of feed supplied and DW waste feed after correcting for dry matter loss to leaching:

(1) AFI=Fi(g)−Fo(g)−(leaching(%)∗(Fi(g)−Fo(g)))

where:

*Fi* (*g*) is dry weight of feed supplied to the vessels (feed in); and

*Fo* (*g*) is dry weight of feed waste from vessels (feed out).

(2) $$leaching=\frac{\left(100\%\right)*\left(Fo\left(g\right)\right)}{Fi\left(g\right)}$$

The AFI is expressed as feed consumed per head per day (g DW*head^−1^*day^−1^).

### 2.4. Analysis of Leaching of Free Amino-Acids (FAAs) and Ninhydrin-Positive Substances 

The leaching of free amino acids (FAAs) and ninhydrin-positive substances from experimental feed was determined using a modified ninhydrin assay [31,32]. Briefly, experimental feeds were immersed in seawater for five time periods (0.5, 1, 3, 6 and 24 h) under the same operating conditions as the experiment in triplicate (n = 3). After immersion, the feeds were siphoned into the separate 124 µm sieves, rinsed with deionised water and frozen for later analysis. The immersed feeds and non-immersed feed samples (t = 0) were freeze-dried until constant weight and ground to a homogenous powder using a hand mortar and pestle. Then, 100 mg samples with 50 ml of deionised (DI) water were homogenised with a hand homogeniser (Cat × 120) for one min each before sonication (Sonics Vibra-Cell CV334) three times for one min and centrifuged (Heraeus Multifuge X1R) for 15 min. Then, 10 µL of supernatant was added to microwell plates, along with 90 µL of DI and 75 µL of ninhydrin reagent (2%). The microwell plates were placed in an oven at 80 °C for 30 min before cooling and the addition of 100 µL of 50% ethanol to stabilise the reaction. Absorbance of samples was measured at 570 nm using a Biotek Synergy HT plate reader. A glycine standard curve was constructed to estimate molar concentrations of FAAs and ninhydrin-positive substances in immersed and non-immersed feed samples.

### 2.5. Growth and Survival

The initial biomass (B_0_) of lobsters was measured for each replicate at stocking of the experiment. Biomass was measured again on days 22 and 42 and at termination of the experiment (day 49). Biomass measurements involved low-impact handling by bulk weighing lobsters in a jug of seawater; the measurements after three and six weeks were used to check progress and adjust the feed ration. At the termination of the experiment, individual lobsters were wet-weighed (WW_t_), and carapace length (CL_t_) was measured (Vernier calliper). The sum of WW_t_ of all survivors in the replicate was used to calculate final biomass (B_t_). Prior to the commencement of the study, individual initial carapace length (CL_0_) and wet weight WW_0_ were measured in three groups of eleven lobsters from the same pool used to stock the experiment by the same procedure. Culture performance parameters were calculated: specific growth rate (SGR), survival (S), average moults per day (AMD), biomass gain (BG), productivity (P), carapace length gain (CLG) and wet weight gain (WWG) [14,30,33]. The analysed parameters were calculated utilizing the following formulae:

(1) WWG = WW_t_ − WW_0_;
(2) CLG (mm) = CL_t_ − CL_0_;
(3) BG (%) = (B_t_ − B_0_)/B_0_ × 100;
(4) SGR (%WW d^−1^) = (lnWW_t_ − lnWW_0_)/t × 100;
(5) AMD (average number of moults *lobster^−1^*day^−1^);
  (3) $$AMD=\frac{X_{R1}+X_{R2}+\cdots +X_{RN}}{R_{N}}$$
  (4) $$X_{R}=\frac{{\left(\frac{N_{R}}{S_{R}}\right)}_{Day\;1}+{\left(\frac{N_{R}}{S_{R}}\right)}_{Day\;2}+\cdots +{\left(\frac{N_{R}}{S_{R}}\right)}_{Day\;49}}{t}$$
(6) P (g m^−3^ day^−1^) = (B_t_ – B_0_) × n_t_/(V × t); and
(7) S (%) = (n_t_/n_0_) × 100

where *X_R_* is the average number of moults × animal^−1^ × replicate^−1^ × day^−1^ (*N_R_*—number of moults in replicate, *S_R_*—number of live animals in replicate), R_N_ is the number of replicates, t is the duration of the experiment (49 d), n is the number of animals and V is the water volume × lobster^−1^ (1.55-L = 0.00155 m^3^). 

### 2.6. Behavioural Observations

Behavioural observations were digitally recorded using time-series photography (GoPro Hero 5 Black cameras) [17,33] during the fifth and sixth weeks of the experiment. Observations were recorded at all tested feeding frequencies and all replicates within each frequency. For each replicate, an image was recorded every 5 s over a 22 h period. To exclude the risk of discrepancies in the analysis of behavioural observations, replicates in which cannibalism occurred during observations were excluded from analysis (two in F16 and one in F1, F4, F8 and F32) due to the increase in lobster activity associated with cannibalism. For equal comparison, analysis was conducted on four random replicates from each tested frequency. Images captured the complete floor area of the experimental tank. The observed area was divided into two sections where activity was monitored: the shelter and the feed sections. Time-series photographs were analysed for all lobsters present in the feeding area. Presence in the feed area was defined as a lobster present in the feed area when food was available, with no physical contact with shelters. A nominal score of one was assigned to each observed lobster present in the feeding area within each recorded photograph. The time that animals spent in the feeding area was calculated in minutes, with each minute of observation comprising twelve images. Image analysis was performed with custom-created MATLAB code. The data for the graphic presentation of behavioural observations were summed into 30 min intervals (preceding 30 min), with the observations of four replicates from one frequency averaged into the 22 h feed presentation timeframe.

### 2.7. MATLAB Code Details

**Figure 2:**
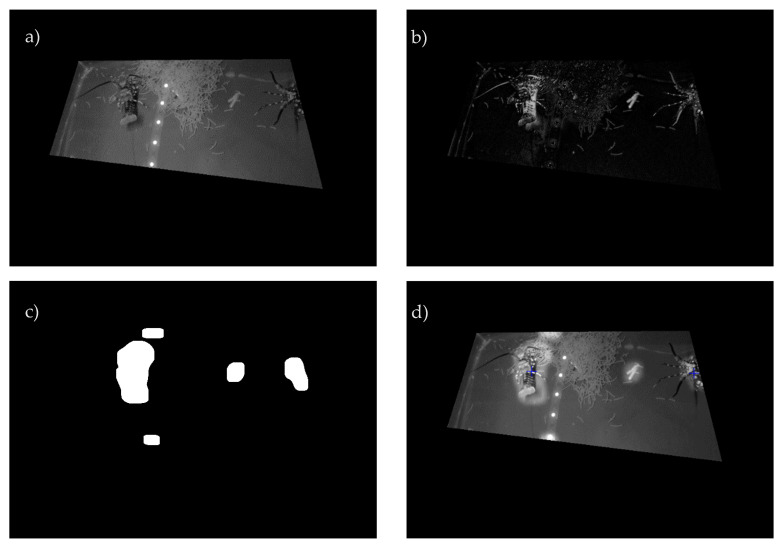
Detection of juvenile lobsters within an aquaculture test tank using adaptive background subtraction. (**a**) Original image with masked region of interest (ROI), (**b**) following background subtraction, (**c**) output of morphological operations identifying candidate detections and (**d**) identification of likely positive detections (blue markers) following statistical vetting of candidates overlayed onto the masked original image with all potential detections highlighted.

Image data were analysed to detect juvenile lobsters within the designated feeding area for each replicate tank. Object detection was performed using an adaptive weighted background subtraction routine to detect areas of movement or significant difference from the generated mean background image (Figure S1). Potential candidate detections were then filtered based on contour size, as well as the mean and standard deviation of a neighbourhood of 37 × 37 pixels centred at the candidate centroid for both original and background images (Figure S1). Finally, the centroid coordinates of likely positive detections were returned (Figure 2). Although not strictly detecting specific objects, the performance of the detection routine was evaluated using standard metrics of recall and precision on a sample of 250 frames of data. Of the 274 ‘ground truth’ lobster instances, 244 true positives (TP) were detected, with 5 false positives (FP) and 35 false negatives (FN), resulting in a recall of 87% and a precision of 98%, where recall = TP/(TP + FN) and precision = TP/(TP + FP).

### 2.8. Statistical Analysis

Statistical analysis of culture performance parameters of lobsters used the six replicate vessels, except for F8, which was analysed using five replicates (as described in Section 2.1). All data were tested for homogeneity of variance with the Shapiro–Wilk W test. When normality was not met, the data were square-root-transformed. Based on the data distribution and type, the statistical analysis included fitting of best regression models to describe the relationship between feed frequency and growth (WW_t_, WWG, CL_t_, CLG, AMD, B and SGR), S and AFI. Behavioural observations expressed as presence in the feeding area were fitted to quadratic polynomial regression and were analysed with two-way ANOVA (phase of photoperiod (light/dark) and feed frequency). When significant difference was found, the Tukey HSD test was performed for post hoc analysis of means. Levene’s test was used for analysis of homogeneity of variances in a two-way ANOVA. To satisfy the homogeneity of variances condition, the data were square-root-transformed prior to analysis. First derivatives of significant quadratic regressions were optimal solutions. The FAA and ninhydrin-positive substance data were fitted to the logarithmic regression. The immersion times of samples tested for FAA included zero values (samples not immersed in water). To account for zero values in the logarithmic regression, all immersion times were increased by 0.0001 h. All data presented in the text show mean value ± S.E. unless otherwise indicated.

## 3. Results

### 3.1. Growth and Survival

**Figure 3:**
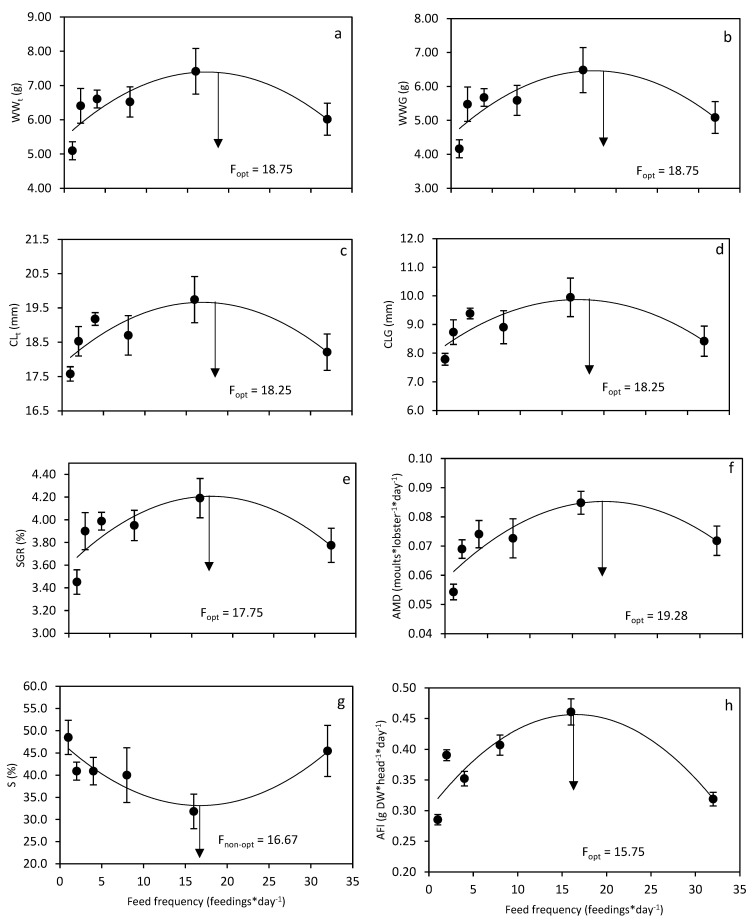
Effect of feed frequency on *Panulirus ornatus* (**a**) final wet weight (WWt), (**b**) wet weight gain (WWG), (**c**) final carapace length (CLt), (**d**) carapace length gain (CLG), (**e**) specific growth rate (SGR), (**f**) average moults per day (AMD), (**g**) survival (S) and (**h**) apparent feed intake (AFI). The graphs show mean values ± S.E. Arrows indicate optimal feed frequencies (Fopt) for WWt, WWG, CLt, CLG, SGR, AMD and AFI. For S, the arrow indicates the non-optimal feed frequency (Fnon-opt).

All growth parameters displayed positive significant quadratic regressions relative to feed frequency (Table 1, Figure 3a–f). According to the quadratic regressions, the highest WW_t_, WWG, CL_t_, CLG, SGR and AMD were found in F16 and the lowest in F1. Calculated optimal feed frequency for WW_t_, WWG, CL_t_, CLG, SGR and AMD ranged between 17.7 and 19.3 feeds day^−1^ (Figure 3a–f). Survival (S) demonstrated a significant negative quadratic regression relative to feed frequency (Table 1, Figure 3g). According to the quadratic regression, the highest S was found in F1and lowest in F16. The calculated minimum S for feed frequency was 16.7 feeds day^−1^. Based on the significant quadratic regressions, B_t_, BG and P did not display significant quadratic relationships relative to feed frequency (Table 1). The highest B_t_ and BG values were found in F4.

### 3.2. Apparent Feed Intake (AFI)

Apparent feed intake (AFI) displayed a significant positive quadratic regression relative to feed frequency (Table 1, Figure 3h). According to the quadratic regression, the highest AFI was obtained in F16. The calculated maximum AFI based on the significant quadratic regression was estimated at a feed frequency of 15.7 feeds per day.

### 3.3. Behavioural Observations

**Figure 4:**
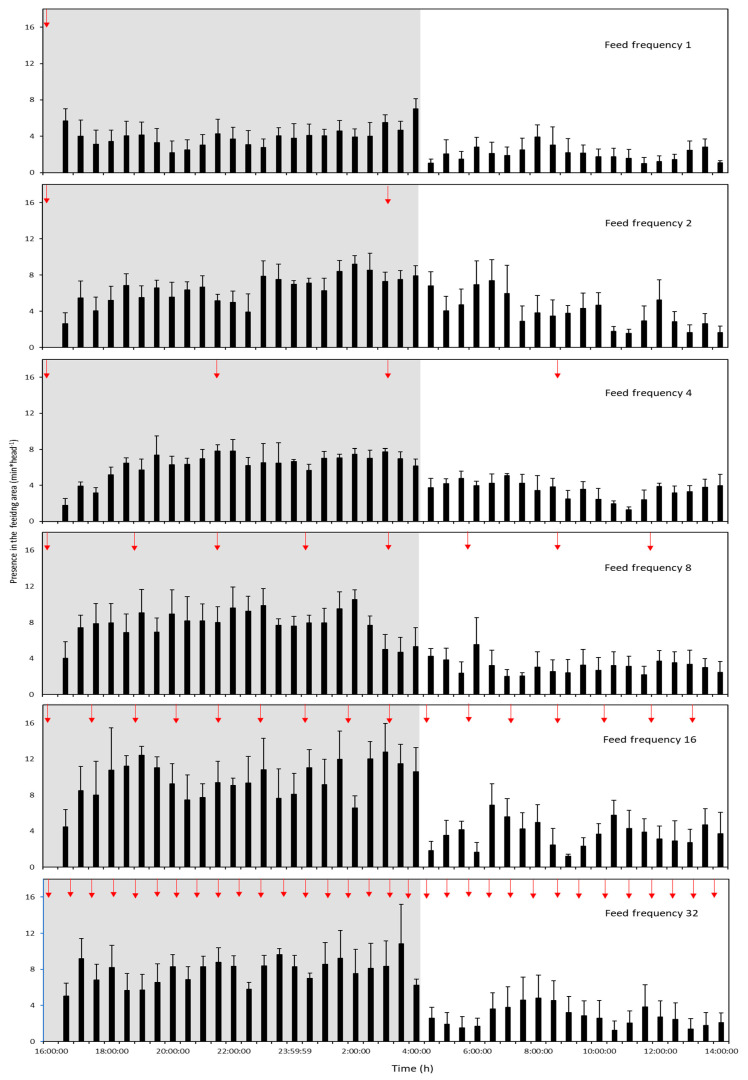
Lobster presence in the feeding area (min × head^−1^) shown for all tested feeding frequencies. Red arrows indicate feed delivery events. Error bars denote S.E.

In all feed frequency treatments, lobsters displayed a general pattern of increased nocturnal activity in the feeding area (Figure 4). Overall observed lobster presence in the feeding area during both the day and night were lowest for F1. Lobster presence in the feeding area appeared to increase through feeding frequencies of F8 and F16 and dropped in F32. The presence of lobsters in the feeding area during daylight was relatively low in all tested frequencies.

**Figure 5:**
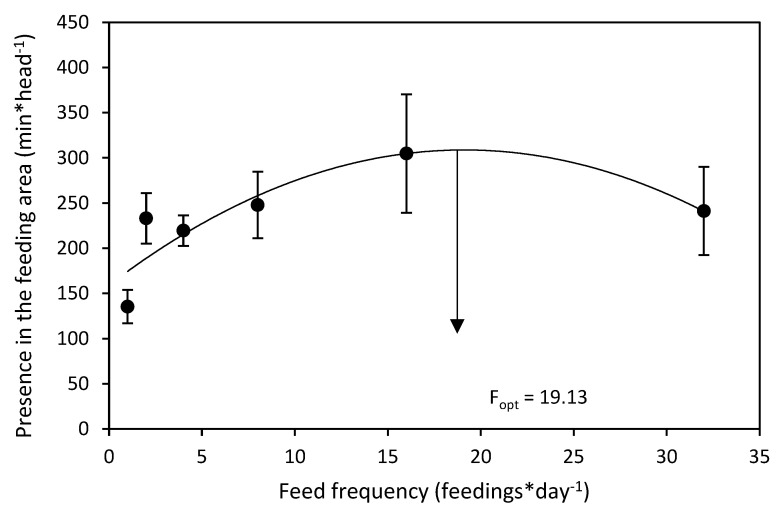
Effect of feed frequency on sum of *Panulirus ornatus* presence in the feeding area. The graph shows mean values ± S.E. The arrow shows optimal feed frequency (F_opt_) for the highest sum of lobster presence in the feeding area.

The total sum of lobster presence in the feeding area during both the day and night showed significant quadratic regression (Table 1, Figure 5). The longest time in the feeding area was shown for F16 and the lowest in F1. The calculated optimal solution based on the significant quadratic regression was 19.1 feeds per day. 

**Figure 6:**
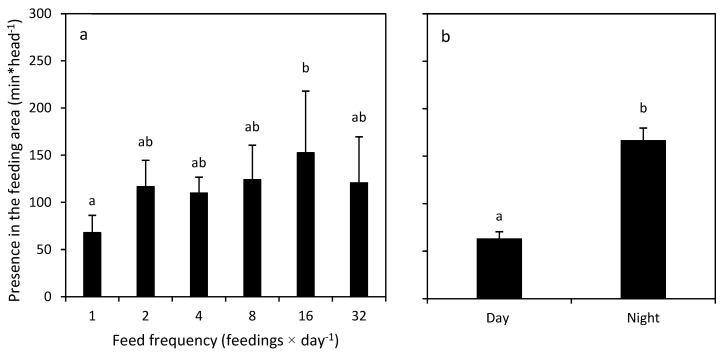
(**a**) Effect of feed frequency and (**b**) daytime on *Panulirus ornatus* presence in the feeding area (min*head^−1^). The graphs show mean values ± S.E., and significant differences (ANOVA) are indicated with superscripts.

In the two-way ANOVA analysis of feeding behaviour, a significant difference was found for feed frequency relative to lobster presence in the feeding area (F = 2.655, d.f. = 5,36, *p* = 0.038). Tukey HSD comparisons showed significant differences between the F1 and F16 feeding treatments (Figure 6). Day/night feeding was also significantly different (F = 68.763, d.f. = 1,36, *p* = 0.000). There were no significant interactions between feed frequency and day/night (F = 1.070, d.f. = 5,36, *p* = 0.393).

### 3.4. Free Amino Acids (FAAs) and Ninhydrin-Positive Substances

**Figure 7:**
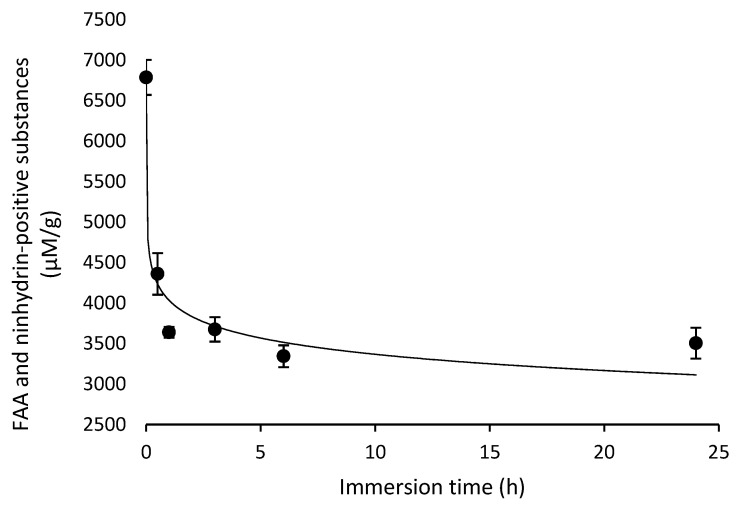
Effect of immersion time on the concentration of free amino acids (FAAs) and ninhydrin-positive substances in the feed. The graph shows mean values ± S.E.

FAAs and ninhydrin-positive substances in the feed displayed a significant negative logarithmic (y = b ln x + a) correlation with immersion time (ANOVA, F = 173.592, d.f. = 1, 16, *p* = 0.000) (Figure 7). The regression describes the influence of immersion time on the concentration of FAAs and ninhydrin-positive substances in the experimental feed, with approximately 53% (from 6,786.9 ± 217.0 to 3,639.3 ± 66.2 µM g^−1^ of the feed, respectively) leached from the feed within the first hour of immersion. Subsequent concentrations of FAAs and ninhydrin-positive substances remained relatively constant at and after three hours of immersion (Figure 7).

## 4. Discussion

A strong relationship was demonstrated between feed frequency and growth, survival, feeding behaviour and feed intake in juvenile *P. ornatus*. The current research indicates the importance of identifying the optimum feed frequency to maximise growth, as well as the negative consequence of some frequencies augmenting cannibalistic behaviour of lobsters at moult and therefore reducing survival. In the context of circadian activity, feed frequency influenced lobster dark-phase activity but not light-phase activity. 

There was a significant effect of feed frequency on juvenile *P. ornatus* growth, which was highest in F16 for all growth parameters. The optimal feeding frequency for growth parameters was found to be between 17.7 and 19.3 feeds day^−1^, suggesting that with this diet, frequent feeding is needed to satisfy the nutritional needs of juvenile *P. ornatus*; this has also been demonstrated for other spiny lobster species [22], penaeids [18,19] and freshwater crayfish [20,34]. The present study is the first in lobsters to show increasing growth, concurrent with increased formulated feed frequency to a peak at F16 and dropping at F32. The peak observed at F16 may be the consequence of omnivorous feeding behaviours, motivating lobsters to be constantly seeking feed in the wild, consuming a range of invertebrates, fish and marine plants [7,8,35]. Such multitrophic feeding indicates that lobsters utilise frequent opportunities to forage in their habitat [7]. The decreased growth at F32 indicates a limit to growth optimisation through the provision of a specific number of feeds per day. Previous studies on spiny lobsters, including *J. edwardsii* and *Panulirus cygnus*, have demonstrated low growth and survival on formulated feeds when compared with control groups fed mussels [30,36,37]. Impediments to effective feed utilisation include feed characteristics, such as attraction, soak times, particle size, manipulation by lobsters and attractant leaching [11,21], as well as lobster biological characteristics, including small foregut capacity, slow filling times and long appetite revival [21,37]. Some of these dietary restraints were addressed by the provision of increased feed frequency compensating for feed attractiveness and leaching properties, as well as potentially compensating for foregut size and filling periods.

Our study confirmed the use of a formulated feed to provide the nutritional requirements for spiny lobsters over a prolonged period of feeding, similar to that used in a prior study by Shu-Chien, Han [38]. In our study, we found that AFI was high, especially when provided at a high frequency (F16). The consistency of our results with the outcomes reported by Shu-Chien, Han [38] suggests that feed, as defined above, sufficiently satisfied the nutritional needs of lobsters. The daily feeding ration in our experiment was set considerably above lobster nutritional needs, at an amount equal to 25% of their WW, whereas daily feed intake has been reported to be as low as 0.8–1.2% of WW [39]. The provision of surplus feed was interpreted to allow the feed ration to be divided into a maximum of 32 feeding events, providing feeding opportunity for all lobsters and minimising cannibalism. However, feeding to excess feed frequency significantly affected growth, survival, feed intake and behaviour. This suggests that frequently motivating lobsters to forage and the regular provision of attractive feed are vital aspects of feed intake optimisation. In this study, the FAAs emitted from experimental feed potentially acted as attractants that stimulated feed intake [16,40]. The ninhydrin method shows positive results for a range of substances, including FAAs, peptides and proteins [41] and demonstrated that FAAs leach quickly from feed under typical aquaculture conditions [42]. The quick leaching of FAAs from feeds is a result of them being low-molecular-weight, water-soluble compounds. Thus, in the present study, the release of FAAs in the first hour provided sufficient attraction to attract lobsters to feed, after which other ninhydrin-positive substances that were stable within the feed were not substantially released and did not enhance feeding behaviour. This suggests that lobsters react readily to the presence of attractive feed and lose interest quickly after depletion of FAA, confirming that attractants are a crucial component of formulated feeds to optimise feed intake and growth in spiny lobster aquaculture [43,44]. 

Important aspects of feeding optimisation include feed detection and discrimination, orientation, approach, start of foraging, continuation or cessation of foraging [45,46]. In this study, the feed attractants stimulated feeding response until leaching limited their effect. With continual exposure to attractants under the F32 frequency, lobsters showed decreased AFI and growth. The doubling of feed frequency from the optimal treatment (F16) may have established a weak but constant background concentration of FAAs in the culture water, resulting in a failure to provide a discrete cue for the provision of fresh feed, either through an insufficient amount or through constant exposure causing habituation. Daniel and Derby [47] noted that lobsters can be habituated to attractants, decreasing their responsivity, which is counterproductive to sustained feed intake. The provision of small feed rations can also be a stimulant for aggressive competition, stress and/or subordination, resulting in decreased feeding activity [11]. 

In contrast to the positive effects of feed frequency of F16 on growth, survival was negatively impacted. This result is intrinsically linked to AMD, with increased moulting frequency providing more opportunities for lobsters to be cannibalised. Similarly, an inverse relationship between nutritionally supported optimum growth and survival was observed in cannibalistic red king crab (*Paralithodes camtschaticus*), which was attributed to increased opportunity for cannibalism as a response to increased moult frequency [48]. During and following ecdysis, soft-shelled lobsters are readily cannibalised by conspecifics, with this complex behaviour defined as killing and eating of conspecifics [49]. In this study, all moults and deceased specimens were at least partly consumed by conspecifics; the only remains found during the recording of moulting and survival. Furthermore, immediate primary cannibalism of moulting lobsters was observed during video observations, which accounted for all of the mortality events during these recording periods. The factors underlying cannibalism include size dependence, aggressive encounters, competition, hierarchy establishment, prolonged moulting [49,50] or, as we observed to be stimulated by the moulting process, commencing immediately before and during ecdysis (Video S1 Cannibalism). The experimental conditions used in this study may have contributed to the possible causes of cannibalism, notably high lobster density, vessel size and animal size distribution. In the wild, cannibalism is a mechanism for regulating density of social groups and handling food scarcity [51]. However, it is acknowledged that increased growth may be related to nutrient supplementation from cannibalism, and it is clear that further research is required to investigate the drivers of cannibalism and its impact on growth in culture. 

Lobsters exposed to F16 were most often present in the feeding area, indicating that *P. ornatus* are likely to forage frequently on attractive feed. This study is the first to demonstrate the effect of feed frequency on the duration of feeding time in spiny lobsters, previously demonstrated in omnivorous crabs (*Pachygrapsus transversus*), which, in their natural environment, foraged 10-fold longer than fish in the same area [52]. During our study, lobsters spent a considerable amount of time in the feed area, with maximum and minimal times of 5 h and 2.3 h for F16 and F1, equalling 23 and 10% of the day (22 h feeding day^−1^), respectively. The remaining frequencies showed lobster presence in the feeding area between 3.7 and 4.2 h, equalling 17.8% of the day. These results are similar to that observed by Do Nascimento, Do Nascimento [53] with freshwater crabs (*Kingsleya attenboroughi*), where they fed for approximately 17% of the day. These observations demonstrate the frequent feeding behaviour of these omnivores and their interaction and selection of a preferred prey when it is available [54]. The frequent feeding behaviour of lobsters is a trait that should be satisfied in aquaculture. 

This study showed a similar presence in the feeding area of *P. ornatus* juveniles during daylight across all treatments. This may be the result of the predator-free environment and learning abilities of spiny lobsters. This observation corresponds with a previous study on *P. argus* juveniles [55]. Lobsters caught from the natural environment remained in shelters during daylight hours for two weeks. After this time, lobsters increased their daytime activity, including feeding and climbing in cages [55]. In juvenile *P. ornatus*, daylight activity may increase through ontogeny in a laboratory environment [33]. This may be an example of the ability of lobsters to assess feeding versus the risk of leaving a shelter during daylight in a culture environment. 

During the experiment, lobsters were observed to be waiting for the start of the feeding cycle near or under the pipe supplying feed. This behaviour was often accompanied by chasing away other lobsters from the feed after delivery, suggesting feed competition and dominance. Feeding competition and agonistic behaviours were observed in *J. edwardsii* exposed to low feed rations and decreased by increasing the feed ration [11]. In this study, increasing feed frequency may have acted to provide attractive feed to all lobsters present in the experimental vessel. After dominant lobsters were satiated, the submissive specimens had an opportunity to feed on attractive feeds delivered later.

## 5. Conclusions

Feed frequency is an important factor that influences AFI, growth, survival and behaviour in *P. ornatus*. The best feed intake and growth occurred at F16, indicating that optimising feed frequency is key to stimulating foraging and improving growth. However, increased growth in F16 consequently increased the likelihood of cannibalism during ecdysis due to higher AMD. Therefore, further research is required to address cannibalism in the aquaculture of *P. ornatus*. Behavioural observations indicate that lobsters start feeding quickly after feed delivery and during the time when feed remains attractive (about 1 h). The constant concentration of attractants maintained in F32 is suspected to be responsible for reduced lobster presence in the feeding area, suggesting a limit with respect to the beneficial effects of multiple feed frequencies.

## Figures and Tables

**Table 1:** Quadratic regression details (y = ax^2^ + bx + c). Quadratic regressions describe the influence of feed frequency on the following parameters: final wet weight (WW_t_), wet weight gain (WWG), final carapace length (CL_t_), carapace length gain (CLG), final biomass (B_t_), biomass gain (BG), specific growth rate (SGR), moulting frequency (AMD), productivity (P), survival (S), apparent feed intake (AFI) and presence in the feeding area. Significant (ANOVA, *p* < 0.05) regressions are marked with an asterisk (*).

Parameter	a	b	c	R^2^	df	F	*P*
WW_t_ (g)	−0.006	0.225	5.474	0.236	2,32	4.934	0.014 *
WWG (g)	−0.006	0.225	4.541	0.236	2,32	4.933	0.014 *
CL_t_ (mm)	−0.006	0.219	17.845	0.226	2,32	4.678	0.017 *
CLG (mm)	−0.006	0.219	8.051	0.226	2,32	4.678	0.017 *
B_t_ (g)	0.011	−0.335	29.197	0.020	2,32	0.333	0.719
BG (%)	0.096	−2.955	175.371	0.018	2,32	0.297	0.745
SGR (% BW d^−1^)	−0.002	0.071	3.601	0.239	2,32	5.031	0.013 *
AMD (moults × lobster^−1^ × day^−1^)	−7.78 × 10^5^	0.003	0.058	0.359	2,32	8.973	0.001 *
P (g m^−3^ day^−1^)	0.031	−1.002	35.588	0.077	2,32	1.327	0.280
S (%)	0.053	−1.767	47.700	0.198	2,32	3.950	0.029 *
AFI (g DW × head^−1^ × day^−1^)	−0.001	0.019	0.301	0.297	2,32	6.759	0.004 *
Presence in the feeding area (min × head^−1^)	−0.409	15.647	159.198	0.265	2,21	3.782	0.040 *

## Data Availability

The data presented in this study are available on request from the first author. The data are not publicly available due to large amount.

## Supplementary Materials

The following supporting information can be downloaded at: https://www.mdpi.com/article/10.3390/ani12172241/s1, Figure S1: Flowchart of main image analysis routine (left) and filtering of potential candidate detections based on pixel intensity statistics of a 37 × 37 pixel region centred at the candidate centroid (right); Video S1: Cannibalism.
