# Supplementary material for: The Effect of Feed Frequency on Growth, Survival and Behaviour of Juvenile Spiny Lobster (Panulirus ornatus)
Source: Animals (Basel). 2022 Aug 30;12(17):2241. doi: 10.3390/ani12172241 (PMC9454674; doi:10.3390/ani12172241)
Supplement: Supplementary file 1 [file animals-12-02241-s001.zip › animals-1842059-supplementary.pdf]

The detection routine flowchart is presented in Figure S1 (left). For each image set, following image pre-processing and re-sizing of images down to 750 x 1000 pixels (0-255 grayscale), the feeding area was manually defined by user input to create a region-of-interest (ROI) mask. Edges of the ROI that bordered hide structures were identified and shifted a set distance of 10 mm to eliminate possible detections of lobsters that were in hides but not active within the feeding area. The resulting ROI mask was applied to the entire image set (Figure 2a). A background image was generated for each image within the set according to the following equation:

$$BI_i = (1 - a) \cdot I_{i-1} + a \cdot BI_{i-1}$$

where BI_i is the background image for the ith image, a is a fixed weighting (a = 0.6), I_{i-1} is the previous image frame, and BI_{i-1} is the previous background image where BI_0 is the pixel-wise mean image of the entire image set. As such the background image was adaptive to account for slight temporal changes within the scene such as the addition or movement of feed pellets. A background subtraction ($I_{i\_BS} = I_i - BI_i$) was then performed (Figure 2b), followed by image binarization, and a series of morphological operations (dilations and erosions) to identify contours of candidate detections (Figure 2c). For each candidate detection the centroid coordinates were calculated and a 37 x 37-pixel neighbourhood was extracted from the original and background images centred at the candidate centroid (N_C and N_B, respectively). Filtering of potential detections was then performed based on contour size, as well as the mean and standard deviation (std) of neighbourhood pixel intensities for both original and background images (Figure S1, right). In reference to Figure S1 (right), fixed values for minimum area (A_{min}), mean (p), standard deviation (q), and ratio of standard deviation values (R) were used for all data (where A_{min} = 300 px, p = 75, q = 10, R = 4). Multiple stages of vetting were performed to account for situations where false positives were likely – surface reflections, movement directly above the feed pile, or feed pellets floating on the tank water surface. Finally, the centroid coordinates of confirmed positive detections were returned (Figure 2d).

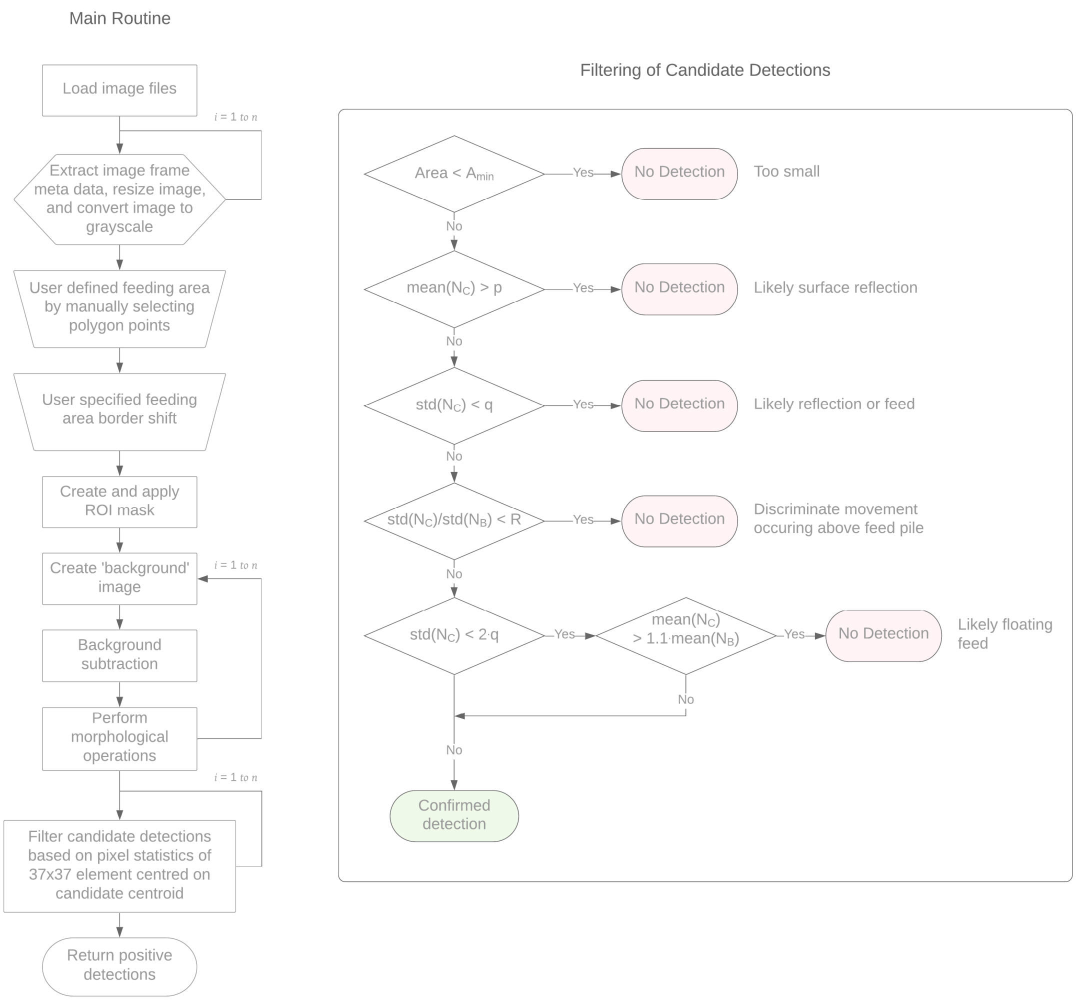

Figure S1. Flowchart of main image analysis routine (left), and filtering of potential candidate detections based on pixel intensity statistics of a 37 x 37 pixel region centred at the candidate centroid (right).
